# Protective Effects of *Fructus Ligustri Lucidi* Fermentation on Fatty Liver Hemorrhagic Syndrome in Laying Hens

**DOI:** 10.3390/ani16142147

**Published:** 2026-07-10

**Authors:** Xiaoli Li, Songchen Yuan, Xixi Li, Xiaowei Ding, Wanling He

**Affiliations:** 1 College of Animal Science and Technology, Henan University of Science and Technology, No. 263 Kaiyuan Road, Luoyang 471000, China; yuansongchen1@163.com (S.Y.); hwling921@126.com (W.H.); 2 Henan Egdoo Technology Group Co., Ltd., No. 221 Lianhua Street, Zhengzhou 450001, China; lixixi0611@163.com (X.L.); dxwxsh@163.com (X.D.)

**Keywords:** *Fructus Ligustri Lucidi*, fatty liver hemorrhagic syndrome, late laying period, Hy-Line brown laying hens, high-energy diet

## Abstract

*Fructus Ligustri Lucidi* (FLL) is a traditional tonic Chinese medicine with a long history of application due to its various pharmacological effects, but there are fewer studies on the use of FLL to alleviate fatty liver in animals. The objective of the current study was to assess the influence of FLL fermentation products on fatty liver hemorrhagic syndrome in hens during the late stages of egg production. The study involved 288 Hy-Line Brown laying hens, aged 500 days, which were randomly allocated into 4 groups, with each group having 6 replicates of 12 chickens. Hens were fed a basal diet (control group, group Ι), a high-energy diet (model group, group II) and a high-energy diet supplemented with 0.05% and 0.1% FLL fermentation product (groups III and IV). The duration of the pre-experiment was 1 week, and the trial period was 12 weeks. The results showed that the addition of FLL fermentation product to high-energy diets significantly increased egg weight and reduced eggshell breakage rate. High-energy diet supplementation with FLL fermentation product increased serum T-AOC and T-SOD activity (*p* < 0.05), and reduced serum MDA content (*p* < 0.05). As more FLL fermentation product was added, there was a notable reduction in lipid droplets in liver tissues, alleviation of symptoms of steatosis, and suppression of mRNA expression levels of lipid synthesis genes *ACC*, *FAS*, and *SCD* in the liver (*p* < 0.001). Transcription factors such as *NR1H3* (*p* < 0.01), *LPK* (*p* < 0.001), and *SREBF1* (*p* < 0.05) were differentially down-regulated, significantly reducing *MTTP* gene expression (*p* < 0.001). It can be concluded that the addition of 0.1% FLL fermentation product can enhance the productivity of laying hens during their late laying phase, increase the antioxidant capacity, inhibit the mRNA expression level of genes related to fat synthesis in the liver, reduce the accumulation of fat, and alleviate the fatty liver of laying hens caused by the high-energy diet.

## Simple Summary

*Fructus Ligustri Lucidi* (FLL), a natural herbal resource, has garnered considerable attention due to its diverse biological activities. Nevertheless, research on FLL’s impact on fatty liver hemorrhagic syndrome (FLHS) has been relatively limited. This study presents data concerning the effects of FLL fermentation on the production performance, serum biochemical parameters, expression of genes related to lipid synthesis, and histopathological alterations in the liver of laying hens during their late laying phase. The findings reveal that FLL fermentation product can enhance the laying rate and egg weight, decrease the eggshell breakage rate, lower serum cholesterol concentrations, boost antioxidant capacity, suppress the mRNA expression of genes involved in lipid synthesis, and improve lipid metabolism in laying hens. These results suggest that FLL fermentation product can ameliorate FLHS in laying hens during the late stages of egg production.

## 1. Introduction

Owing to its high quality and affordable price, eggs are a regular part of our daily food consumption. Nonetheless, the poultry industry faces a significant hurdle in the form of fatty liver hemorrhagic syndrome (FLHS), which commonly affects laying hens. FLHS, a metabolic disorder, disrupts the creation and movement of low-density lipoprotein and the removal of fatty acids from the liver, causing issues in lipid metabolism and an overabundance of lipids in the liver [1]. Notably, FLHS adversely affects the production performance and health of laying hens, particularly those in good body condition and housed in cages. It may lead to a rapid drop in egg production and reduce the duration of the peak egg-laying phase [2]. Furthermore, a previous study has demonstrated that FLHS is the primary cause of non-infectious mortality in caged laying hens [3]. Whether directly or indirectly, FLHS inflicts substantial economic losses on farmers and the poultry industry as a whole. Therefore, it is crucial to identify an effective treatment to mitigate the damage caused by FLHS.

Despite advancements in understanding the pathogenesis of FLHS, therapeutic options remain limited. The pathogenesis of FLHS remains inadequately understood; however, the “two-hit” hypothesis is a widely accepted explanatory model. The initial “hit” involves the excessive accumulation of triglycerides in the liver and the inhibition of fatty acid oxidation, leading to the disruption of lipid metabolism homeostasis. Subsequently, oxidative stress and insulin resistance generate excessive reactive oxygen species in the liver, exacerbating the degradation of reticulin fibers, which constitutes the second “hit” [4]. Consequently, the use of antioxidants may mitigate the incidence of FLHS. Medicinal herbs, renowned for their disease-treating properties, serve as vital natural sources of novel bioactive compounds. *Ligustrum lucidum* Ait, a medicinal plant, widely distributed in China, is one such example. Its fruit, known as FLL, is a well-known traditional Chinese medicine. According to traditional Chinese medicine records, FLL helps maintain healthy energy, nourishes the liver and kidneys, and strengthens bones [5]. Due to its diverse pharmacological effects, FLL has a long history of use in traditional Chinese medicine, including antioxidant and anti-inflammatory properties. Modern research has revealed that FLL extracts and compounds exhibit a broad spectrum of pharmacological effects, including antitumor, liver-protective, hypoglycemic, lipid-lowering, and immune-regulatory properties [6]. However, there is a lack of information regarding the interactive effects of dietary FLL on hepatic fat metabolism in laying hens, particularly in aged ones. This study aims to offer effective nutritional intervention strategies for preventing or alleviating FLHS by incorporating FLL fermentation products into the diet.

## 2. Materials and Methods

### 2.1. Preparation of Experimental Materials

The study utilized 288 Hy-Line Brown laying hens, all 500 days old and of similar weight (a laying hen weighs between 2.20 and 2.30 kg), sourced from local commercial poultry farms in Henan Province (Luoyang, China). Additionally, FLL was procured from a pharmacy in Luoyang, Henan Province, China, and finely ground into powder using a knife mill before undergoing fermentation at the Microbiology Laboratory of Henan University of Science and Technology.

### 2.2. Analysis of Ligustrum lucidum Chemical Composition Before and After Fermentation

In this study, Bacillus licheniformis was employed for the solid-state aerobic fermentation of FLL. The fermentation parameters were determined based on optimal conditions identified in prior research conducted by our laboratory, which include a bacterial inoculum concentration of 12%, a substrate moisture content of 48%, a fermentation temperature of 37 °C, and a fermentation duration of 42 h.

One gram of each sample of FLL, both pre- and post-fermentation, was precisely weighed and placed separately in capped glass tubes. These samples were then mixed with an appropriate volume of 70% methanol solution and soaked for 30 min to ensure thorough wetting of the plant material. Subsequently, the tubes containing the samples were immersed in an ultrasonic water bath and subjected to irradiation at the predetermined extraction temperature and duration. The samples were centrifuged at 9600 rpm for 10 min post-extraction, and the supernatants obtained were collected and diluted for High Performance Liquid Chromatography (HPLC) analysis. The Sep-Pak C18 solid-phase extraction column was employed, utilizing mobile phases A and B, which consisted of 0.1% trifluoroacetic acid and 80% acetonitrile, respectively. Prior to use, the solutions were filtered through a 0.22 μm membrane and degassed using ultrasound. A sample volume of 10 μL was introduced, with the elution flow rate maintained at 1.0 mL/min. Absorbance measurements were conducted using a UV detector set at a wavelength of 280 nm.

### 2.3. Animals, Diets, and Experimental Design

The present study was carried out at the Experimental Ranch, located within Henan University of Science and Technology in Luoyang, Henan Province, China. Ethical approval for the study was obtained from the Animal Ethics Committee of Henan University of Science and Technology (Approval No. HAUST-026-P0513082). A total of 288 Hy Line Brown chickens (all 500 days old) participated in a one week pilot period and a 12 week formal trial. Prior to the trial, the hens were fed a corn-based basal diet for two weeks to facilitate adaptation and achieve a uniform egg production rate. The hens were randomly split into four groups, each containing six replicates of 12 birds housed in four adjacent cages with three birds per cage, following an additional week of adaptation in cages measuring 70 cm by 30 cm by 40 cm. Before the experiment commenced (day 0), no significant differences were observed in egg production rate or egg weight across the four groups (*p* > 0.05). Birds were evenly allocated to upper and lower cages within each replicate group to lessen cage-level impacts. The study involved four treatment groups: Group I (CON) received a standard diet; Group II was given a high-energy diet (HE, with a metabolizable energy of 11.92 MJ/kg) to establish a pathological fatty liver model [7]; and Groups III and IV were provided with the same high-energy diet as Group II, but with FLL fermentation product supplementation at levels of 0.05% and 0.1%, respectively. The detailed composition of the basal and HE diets is presented in Table 1. To meet the nutritional demands of laying hens, the basal diet was developed following the guidelines of NRC (1994) and NY/T (33-2004) [8,9]. Throughout the 12-week experiment, the hens’ diets were newly prepared biweekly, and they had free access to food and water while being subjected to a 16 h light and 8 h dark cycle. All procedures in the experiment received approval from the Institutional Animal Care and Use Committee of Henan University of Science and Technology, China. Egg production, egg weight and cracked-eggs were recorded daily throughout the experimental period, and laying rate (including cracked eggs) and cracked-egg rate were calculated accordingly.

### 2.4. Sample Collection

Upon completing the experiment, 12 hens from each group (two from each replicate) were randomly chosen and euthanized to obtain blood and liver samples for analyzing serum biochemical parameters. For mRNA expression analysis, liver samples were prepared separately, promptly placed in liquid nitrogen, and then transferred to a −80 °C deep freezer.

### 2.5. Blood Biochemistry and Histopathological Analysis

After collecting blood from the wing vein in vacutainer serum tubes, the samples were centrifuged at 3000 rpm for 15 min to extract serum, which was then preserved at −20 °C for future analysis. Blood biochemistry indices in the serum, including total cholesterol (TCH, Cat. No. A111-1-1), triglyceride (TG, Cat. No. A110-2-1), high-density lipoprotein cholesterol (HDL-C, Cat. No. A112-2-1), and low-density lipoprotein cholesterol (LDL-C, Cat. No. A113-2-1), were assessed using the COBUS MIRA Plus automatic analyzer (Roche Diagnostic System Inc., Rotkreuz, Switzerland) based on the manufacturer’s instructions, as well as serum enzyme activity indicators, including alanine aminotransferase (ALT, Cat. No. C009-2-1) activity, and aspartate aminotransferase (AST, Cat. No. C010-2-1) activity, were measured using ELISA assay kits obtained from Nanjing Jiancheng Bioengineering Institute Co., Ltd. (Nanjing, China), following the manufacturer’s instructions. Liver tissue samples were prepared and stained with HE and oil red O following standard protocols to observe pathological changes, and the slides were examined using a Leica DMI6000 140 B microscope from Leica Co., Ltd., Wetzlar, Germany.

### 2.6. Determination of Antioxidant Index

Serum antioxidant indicators, such as total antioxidant capacity (T-AOC, Cat. No. A015-2-1), total superoxide dismutase (T-SOD, Cat. No. A001-3-2) activity, glutathione peroxidase (GSH-Px, Cat. No. A005-1-2) activity, catalase (CAT, Cat. No A007-1-1) activity, and malondialdehyde (MDA, Cat. No. A003-1-2) content were measured using ELISA assay kits purchased from Nanjing Jiancheng Bioengineering Institute Co. Ltd. (Nanjing, China), following the manufacturer’s protocols.

### 2.7. Quantitative Reverse Transcription Polymerase Chain Reaction (qRT-PCR) Analysis

Trizol reagent from Tsingke Biotech (Beijing, China) was used to extract total RNA from the collected liver samples. The concentration of RNA was determined using a NanoDrop 2000 spectrophotometer from Thermo Fisher Scientific (New York, NY, USA), and the purity of RNA was checked by running it on a 1% agarose gel through electrophoresis. Subsequently, complementary DNA (cDNA) was synthesized from the total RNA using the ToloScript RT EasyMix kit from Tsingke Biotech (Beijing, China). The primers of the selected genes (i.e., acetyl-CoA carboxylase, *ACC*; fatty acid synthase, *FAS*; stearoyl-CoA desaturase, *SCD*; carbohydrate response element binding protein, *ChREBP*; L-type pyruvate kinase, *LPK*; microsomal triglyceride transfer protein, *MTTP*; sterol regulatory element binding protein 1, *SREBF1*; nuclear receptor subfamily 1 group H member 3, *NR1H3*) for fatty acid synthesis were produced. The sequences of the primers are provided in Table 2. SYBR Green Master Mix kits (Tsingke Biotechnology Co., Ltd., Beijing, China) were used for RT-qPCR on a Bio-Rad CFX96 platform (Bio-Rad, Richmond, CA, USA), following the manufacturer’s instructions. The PCR cycle conditions were as follows: initial denaturation at 95 °C for 30 s, followed by 40 cycles of denaturation at 95 °C for 15 s, annealing at 60 °C for 30 s, and extension at 60 °C for 30 s. The β-actin gene, serving as a housekeeping gene, acted as an internal control. The 2^−ΔΔCT^ method was utilized to normalize the relative mRNA expression levels of the target genes to this control [10].

### 2.8. Statistical Analysis

One-way analysis of variance (ANOVA) was used for statistical analyses with SPSS version 27.0, and Duncan’s multiple range test was employed for multiple comparisons. The data were reported as the mean and standard error, with a statistical significance threshold of *p* < 0.05. Data visualization was performed using Origin 2018 software. The area of the lipid droplets was calculated using ImageJ (1.8.0).

## 3. Results

### 3.1. Analysis of Components in Fermented Ligustrum lucidum

This research employed ultrasound-assisted technology in conjunction with HPLC equipped with a photodiode array detector to assess the concentrations of oleanolic acid, ursolic acid, and total flavonoids in *Ligustrum lucidum*, both prior to and following fermentation. The concentrations of oleanolic acid, ursolic acid, and total flavonoids prior to and following fermentation were 7.490 mg/g versus 8.31 mg/g, 1.607 mg/g versus 2.30 mg/g, and 78.142 mg/g versus 94.02 mg/g, respectively. Post-fermentation, the concentrations of these compounds exhibited increases of 10.95%, 42.86%, and 26.81%, respectively, compared to their pre-fermentation values.

### 3.2. The Impact of FLL Fermentation Product on Pathological Alterations and Lipid Accumulation in Hepatic Cells

**Figure 1:**
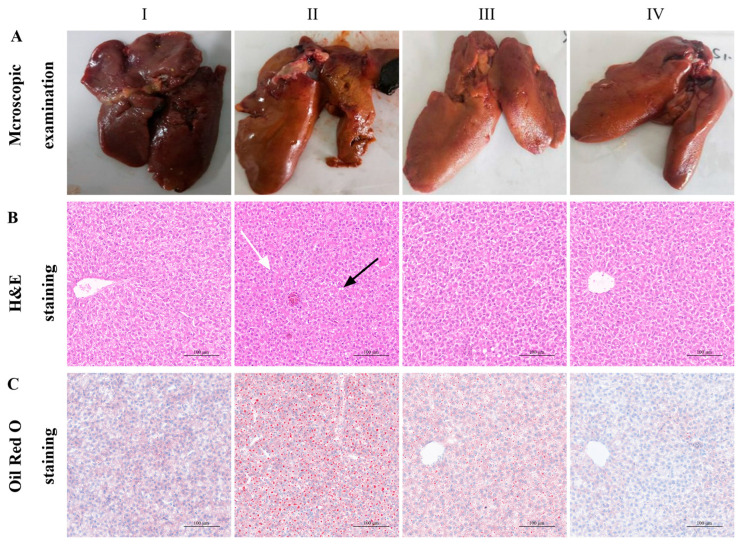
Pathological observation results of the liver. I: basal diet group, II: high-energy diet group, III: high-energy diet + 0.05% FLL fermentation product group, IV: high-energy diet + 0.1% FLL fermentation product group. (**A**) Histopathological observation. (**B**) H&E staining, Scale bar, 100 µm. The black arrow in the figure points to lipid droplet vacuoles, and the white arrow points to nuclear pyknosis. The image is magnified 400 times. (**C**) Oil Red O staining, Scale bar, 100 µm. The image is magnified 400 times.

The results of the pathological examination of liver tissue are presented in Figure 1. The findings indicate that, in comparison to the control group, the liver of the experimental group II exhibited a yellow-brown coloration, greasy texture, dull and rounded edges, and a few hemorrhagic spots on the surface. These characteristics align with the symptoms of naturally occurring FLHS, thereby confirming the successful establishment of the FLHS model in this study. When the FLL fermentation product was administered at a concentration of 0.05%, the liver remained fragile and yellow-brown in appearance. Conversely, the introduction of 0.1% FLL fermentation product resulted in a notable alleviation of typical FLHS symptoms (Figure 1A). Additionally, the results of Hematoxylin and Eosin (HE) and Oil Red O staining corroborated these observations. As illustrated in Figure 1B,C, the liver cell architecture in the control group appeared clear and normal, whereas the liver of experimental group II laying hens exhibited severe fatty degeneration, characterized by the accumulation and necrosis of lipid droplets. In comparison to experimental group II, laying hens supplemented with FLL fermentation products exhibited a decrease in liver lipid accumulation induced by high-energy feeding, as evidenced by the lipid droplet area measurements (Group I: 0.386 µm^2^, Group II: 227.083 µm^2^, Group III: 45.910 µm^2^, Group IV: 1.543 µm^2^). These findings consistently suggest that FLL fermentation products have the potential to ameliorate liver steatosis caused by high-energy feeding in laying hens.

### 3.3. Egg Production Performance

Table 3 presents a comprehensive analysis of the egg production performance of laying hens subjected to various dietary treatments. Relative to the control group, both the laying rate and egg weight exhibited significant reductions (*p* < 0.05), while the egg breakage rate was observed to be highest in the group fed the HE diet (*p* < 0.05). Notably, the laying rate was significantly elevated in the cohort receiving a 0.1% FLL fermentation product compared to the HE-diet group. Moreover, egg weight was significantly greater in the groups administered 0.05% and 0.1% FLL fermentation product than in both the control and HE-diet groups (*p* < 0.001), and the egg breakage rate was significantly lower than that recorded in the HE-diet group (*p* < 0.001).

### 3.4. Serum Biochemical Indicators

As depicted in Table 4, there was a significant increase in LDL-C, TG, TCH, and ALT concentrations in laying hens fed the HE-diet in the model group when contrasted with the control (*p* < 0.05). The FLL fermentation product group exhibited significantly reduced serum concentrations of LDL-C, TG, TCH, and ALT in comparison to the model group (*p* < 0.05). However, in the 0.05% FLL fermentation product treatment group, significantly higher concentrations of LDL-C and ALT were observed compared to the control group (*p* < 0.01). The concentrations of LDL-C, TG, TCH, ALT, and AST did not significantly differ between the control group and the 0.1% FLL fermentation product treatment group. There were no major variations in HDL-C concentrations across the experimental groups. The results suggest that the 0.1% FLL fermentation product treatment group demonstrated a more pronounced effect in reversing biochemical blood indicators compared to the 0.05% treatment group.

### 3.5. Serum Antioxidant Indicators

Table 5 presents the serum antioxidant indicators for laying hens across different dietary treatment groups. In comparison to the control group, the concentrations of serum T-SOD and T-AOC were notably reduced in the HE-diet group of laying hens, while the serum MDA concentration was significantly elevated. The serum T-SOD and T-AOC concentrations in the 0.05% and 0.1% FLL fermentation product treatment groups were considerably elevated compared to the HE-diet group, whereas the MDA concentration was considerably decreased. Relative to the control group, the FLL fermentation product treatment group of laying hens did not exhibit significant differences in serum T-SOD, GSH-Px, CAT, MDA, and T-AOC concentrations.

### 3.6. The Effects of FLL Fermentation Product on Metabolism-Regulating Factors in the Liver

**Figure 2:**
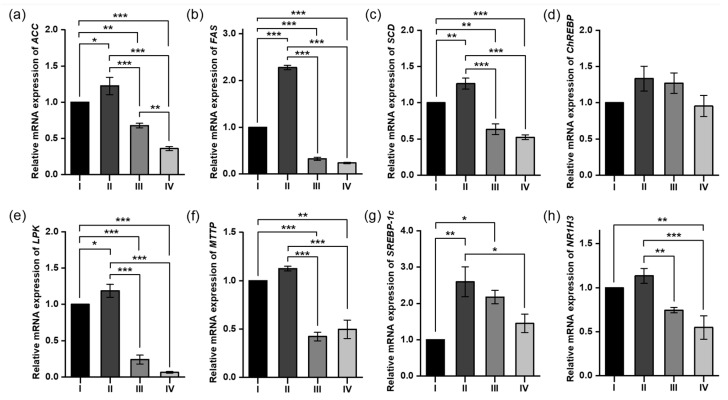
Effects of different dietary treatments on the relative expression of mRNA of lipid metabolism-related genes in liver tissues of laying hens. I: basal diet group, II: high-energy diet group, III: high-energy diet + 0.05% FLL fermentation product group, IV: high-energy diet + 0.1% FLL fermentation product group. Data are presented with the means ± SEM. * indicates a statistically significant difference between the two groups; * *p* < 0.05; ** *p* < 0.01; *** *p* < 0.001. (**a**) *ACC* = Acetyl-CoA carboxylase; (**b**) *FAS* = Fatty Acid Synthase; (**c**) *SCD* = Stearoyl-CoA desaturase; (**d**) *ChREBP* = Carbohydrate response element binding protein; (**e**) *LPK* = L-type pyruvate kinase; (**f**) *MTTP* = Microsomal triglyceride transfer protein; (**g**) *SREBF1* = Sterol regulatory element binding protein-1c; (**h**) *Nr1h3* = Nuclear receptor subfamily 1 group H member 3.

In laying hens, the liver serves as the primary organ for fat metabolism. This study examined the expression levels of genes associated with lipid metabolism in the liver, as depicted in Figure 2. Relative to the control group, the high-energy diet group exhibited a significant upregulation in the mRNA expression levels of lipid synthesis genes, including *ACC* (*p* < 0.05), *FAS* (*p* < 0.001), *SCD* (*p* < 0.01), as well as the transcription factors *LPK* (*p* < 0.05) and *SREBF1* (*p* < 0.01). In contrast, the 0.05% FLL fermentation product group demonstrated a significant downregulation in the relative mRNA expression levels of *ACC* (*p* < 0.01), *FAS* (*p* < 0.001), *SCD* (*p* < 0.01), *LPK* (*p* < 0.001), and *MTTP* (*p* < 0.001) compared to the control group. Similarly, the 0.1% FLL fermentation product group showed significant downregulation in the expression of *ACC* (*p* < 0.001), *FAS* (*p* < 0.001), *SCD* (*p* < 0.001), *LPK* (*p* < 0.001), *MTTP* (*p* < 0.01), and *NR1H3* (*p* < 0.01) genes. When compared to the high-energy diet group, the inclusion of 0.05% and 0.1% FLL fermentation products led to a significant downregulation in the relative mRNA expression of *ACC*, *FAS*, *SCD*, *LPK*, and *MTTP* (*p* < 0.001), with a differential downregulation observed in the transcription factor *NR1H3* (*p* < 0.01). Furthermore, the application of 0.1% FLL fermentation product resulted in a statistically significant reduction in the relative mRNA expression of *SREBF1* (*p* < 0.05). In contrast, no significant differences were observed in the transcriptional levels of the gene encoding *ChREBP* across all experimental groups.

## 4. Discussion

In laying hens, FLHS is mainly marked by an overabundance of lipids in the liver, leading to severe steatosis accompanied by hepatic hemorrhage. This condition often manifests as increased body weight, abdominal obesity, and a marked reduction in egg production rates among affected birds [11]. FLHS is known to be a major cause of death in commercial laying hens and is acknowledged as the leading noninfectious reason for mortality in these birds, with caging practices exacerbating overall mortality due to FLHS [12]. The establishment of an FLHS model in laying hens is crucial for investigating the pathogenesis of this disease and potential prevention and control measures. Several methods have been reported for establishing an FLHS model in laying hens, including exogenous injection of oestradiol benzoate [13] and adjustment of dietary ratios [14,15]. However, due to its simplicity and ease of implementation, feeding hens a high-energy, low-protein diet is currently the most common method for establishing an FLHS model in laying hens. The pathogenesis, disease progression, and onset of symptoms in hens from this FLHS model closely resemble those of naturally occurring FLHS in production practice. In this study, we established an FLHS model by feeding laying hens a high-energy, low-protein diet. We observed hepatomegaly and marked signs of haemorrhage in the FLHS model laying hens. Furthermore, the livers were fragile and brittle. Histological analysis of the livers from FLHS laying hens revealed hepatocyte destruction, blurred hepatic trabeculae, and an increased number of lipid vacuoles, indicating marked steatosis. ALT concentrations in the livers of FLHS laying hens were significantly elevated, indicating the successful establishment of the FLHS model in laying hens. The modelling method used in this study to induce FLHS in laying hens, as well as the evaluation system for determining the validity of the model based on liver pathological characteristics, are fully consistent with the research protocol previously reported by Wang Nannan’s team [16].

The primary characteristics of FLHS include dysregulation of blood lipids and hepatic steatosis, which can result in elevated concentrations of TG, TCH, and LDL-C, together with a drop in HDL-C and heightened lipid storage in the liver [17]. ALT and AST serve as critical biochemical markers of liver function, reflecting the extent of hepatic damage [18]. HDL-C and LDL-C, as lipoproteins, are crucial in the absorption and movement of lipids and cholesterol throughout the blood, liver, and tissues [19]. It has been demonstrated that improving blood lipid profiles, such as reducing TG, TCH, and LDL-C concentrations while increasing HDL-C, aids in mitigating liver lipid accumulation and damage in laying hens affected by FLHS [20]. Notably, the biochemical and pathological analyses conducted in this study revealed that treatment with the FLL fermentation product ameliorated blood lipid disorders and pathological liver alterations in laying hens subjected to a HE diet. Furthermore, the concentrations of ALT, TG, TCH, and HDL-C were modulated by the FLL fermentation product. In addition, in the groups treated with the FLL fermentation product, the results of blood biochemistry and pathological liver changes were consistent, suggesting the ameliorative effects of the FLL fermentation product on FLHS.

Excessive fat deposition has been shown to compromise the antioxidant system in laying hens, leading to an overproduction of reactive oxygen species (ROS) [21]. Within the antioxidant defense system, T-SOD serves as the primary mechanism for converting superoxide radicals into hydrogen peroxide, which is subsequently transformed into water by CAT or into non-toxic hydroxyl compounds by GSH-Px, utilizing glutathione as a reductant [22]. In cases where antioxidants, including T-SOD and GSH-Px, fail to sufficiently counter excessive ROS generation, homeostasis is disrupted, resulting in oxidative stress that can further exacerbate FLHS [21,23]. MDA is a byproduct of hepatic lipid peroxidation, wherein free radicals within organisms interact with lipids, initiating peroxidation reactions. The resultant oxidation product, malondialdehyde, is known to induce cross-linking and polymerization of essential macromolecules such as proteins and nucleic acids, thereby exhibiting cytotoxic properties. The concentration of MDA serves as an indicator of the degree of lipid peroxidation and cellular damage in organisms. Previous studies have demonstrated that FLL can improve the concentrations of antioxidant enzymes such as SOD, CAT, and GSH-Px, while concurrently reducing MDA concentrations in the serum or liver in poultry [24,25,26,27]. Based on these findings, we hypothesized that FLL might augment antioxidant enzyme activity, mitigate oxidative stress, and prevent FLHS in laying hens. Consistent with our hypothesis, the present study observed a marked decrease in serum total SOD and GSH-Px activities, alongside a significant increase in MDA content within the HE group. Conversely, supplementation with the FLL fermentation product significantly enhanced T-AOC and total SOD activity, while reducing MDA content. These results suggest that the FLL fermentation product may alleviate FLHS in laying hens by scavenging free radicals, inhibiting lipid peroxidation, and enhancing antioxidant enzyme activity. This study exclusively examined oxidative stress markers (T-SOD, GSH Px, CAT, MDA, and T-AOC) in serum samples. Measuring these markers in liver tissue homogenates would offer more direct experimental evidence.

The transcriptional regulation of lipogenesis involves crucial transcription factors like *NR1H3, SREBF1,* and *ChREBP. NR1H3* and *SREBF1* are integral to the regulation of lipogenesis in response to insulin, whereas *ChREBP* is primarily involved in glucose-induced lipogenesis [28,29]. ACC and FAS serve as critical rate-limiting enzymes in lipid synthesis. There is a positive correlation between *FAS* mRNA expression levels and body fat content in animals [30], with the synthesis of fatty acids in tissues being largely determined by *FAS* mRNA expression levels [31]. *SREBF1*, a member of the nuclear transcription factor family, regulates lipid synthesis in vivo by modulating enzymes related to lipid metabolism, such as ACC, FAS, and SCD. It has been demonstrated that hepatic lipid synthesis can be attenuated by inhibiting the activity of SREBF1 and its downstream targets, including ACC, FAS, and SCD, in both poultry and mammals [32]. Moreover, research has demonstrated that ACC inhibitors effectively suppress hepatic lipogenesis, mitigate hepatic steatosis, and hold promise as therapeutic agents for non-alcoholic fatty liver disease [33]. ChREBP is a protein that specifically binds to a carbohydrate response element within the promoter region of the *LPK* gene, thereby regulating the transcription of genes involved in glycolysis and lipogenesis [34,35].

According to Yang et al. [36], FLL markedly decreased the expression levels of *SREBF1* and *NR1H3* mRNA in the liver. Furthermore, oleanolic acid has been shown to reduce the expression of cholesterol regulatory element-binding proteins and significantly diminish the promoter activity of *NR1H3* response elements and *SREBF1.* This subsequently causes a decline in the expression of mRNA and proteins associated with *NR1H3* target genes, thereby reducing lipid storage in hepatocytes [37]. Additionally, salicin in FLL down-regulates the expression of *SREBP* genes (*SREBP-1c* and *SREBP-2c*), and concurrently suppresses the expression of *SREBP* target and downstream genes, such as those encoding *FAS* and *SCD* [38]. The results of this study are consistent with earlier research, demonstrating that the fermentation product of FLL significantly down-regulates the expression of *NR1H3* and *MTTP,* along with the target genes *ACC, FAS,* and *SCD* in the liver. Given the observed gene expression patterns of *ACC, FAS*, and *NR1H3*, it is hypothesized that the dietary inclusion of FLL fermentation product may reduce serum triglyceride levels by diminishing fatty acid synthesis, as indicated by the down-regulation of *ACC, FAS*, and *SCD* expression. This, in turn, could lower the risk of hepatic damage.

FLHS has been documented to significantly impair egg production and reduce feed intake in laying hens [39]. Walzem et al. [40] demonstrated that laying hens with over-induced FLHS exhibited irregular egg production, an increased incidence of double ovulation, shell defects, follicular collapse, and oviductal degeneration. Consistent with previous findings, the present study observed a significant reduction in both egg production rate and egg weight, alongside a notable increase in the egg breakage rate among FLHS-affected laying hens. The primary chemical constituents of FLL include triterpenoids, iridoids, flavonoids, and phenylethanol glycosides. The experimental results indicated that incorporating 0.1% FLL fermentation product into the high-energy diet exerted a beneficial effect by enhancing egg production and egg weight while reducing the egg breakage rate. This effect is hypothesized to be attributable to the total flavonoids present in FLL. Flavonoids, as a class of phytohormones, including glycosides and methylated derivatives, have the capacity to influence life-regulating processes in animals. Huang et al. [41] demonstrated that the dietary inclusion of mulberry leaf flavonoids enhances the antioxidant capacity and calcium deposition in the uterine shell glands of aged laying hens, leading to increased eggshell thickness and strength, as well as a reduction in the egg breakage rate. Similarly, Guo et al. [42] observed that epimedium flavonoids significantly enhance the proliferation and differentiation of granulosa cells within the follicles of laying hens, thereby promoting follicular development and increasing egg production rates. Given their compositional similarity to these phytoflavonoids, FLL flavonoids may potentially enhance ovarian function and egg production by augmenting follicular proliferation and differentiation while reducing apoptosis in laying hens.

In addition to its regulatory role in follicular development, enhanced liver function may constitute a principal mechanism through which fermented FLL augments egg-laying performance. The liver serves as the central organ for lipid metabolism and the synthesis of yolk precursors in poultry, being responsible for the synthesis and transport of yolk precursors, such as very low-density lipoproteins and ovovitelin, to the ovaries. Under conditions of FLHS, the abnormal accumulation of lipids within hepatocytes not only results in structural damage to the liver and a decline in metabolic function but also adversely affects the efficiency of nutrient conversion and utilization. This, in turn, restricts the availability of nutrients necessary for yolk formation and egg production. Previous research has demonstrated that hepatic steatosis impairs both lipid export capacity and protein synthesis, thereby negatively impacting the reproductive performance of laying hens. In this study, fermented FLL significantly reduced hepatic lipid deposition, improved histopathological damage to the liver, and lowered the levels of serum abnormalities in liver function-related indicators, suggesting that hepatic metabolic function had been restored. As hepatic lipid accumulation decreased, more nutrients were released from abnormal storage states and reallocated to physiological processes related to egg production. Concurrently, the restoration of hepatocyte function helps to enhance the efficiency of fatty acid oxidation, lipoprotein assembly, and the synthesis and transport of yolk precursors, thereby providing adequate nutritional support for follicular development and yolk deposition. Furthermore, the reduction in hepatic inflammation and oxidative stress levels also helps to minimise the energy expenditure required to maintain homeostasis, allowing more metabolic resources to be prioritised for the maintenance and enhancement of production performance [43]. Consequently, the increase in egg production observed in this study may stem not only from improvements in reproductive system function, but is more likely to be closely related to reduced lipid accumulation in the liver, enhanced metabolic capacity, and improved efficiency of nutrient allocation. These findings further illustrate that alleviating the hepatic dysfunction caused by FLHS is of significant importance for restoring the production performance of laying hens, and also suggest that fermented FLL may indirectly promote improved egg production by enhancing liver health [44].

This study demonstrates that fermentation products derived from FLL exert significant effects in ameliorating fatty liver, as evidenced by a reduction in hepatic lipid accumulation, mitigation of pathological damage to liver tissue, and improvement in associated biochemical markers. We hypothesize that these effects may result from the synergistic interactions of active constituents within the FLL fermentation products. The process of microbial fermentation disrupts plant cell wall structures, facilitating the release of bioactive compounds such as phenols, flavonoids, and terpenes, while converting macromolecular components into smaller metabolites that are more readily absorbed and utilized, thereby enhancing their bioavailability and biological efficacy [45]. Notably, the active constituents in FLL, including oleanolic acid, ursolic acid, and total flavonoids, exhibit lipid-lowering, antioxidant, and hepatoprotective properties. Post-fermentation, the concentration of these active substances is significantly elevated, which may account for their enhanced capacity to modulate disorders of hepatic lipid metabolism [44].

Furthermore, the initiation and progression of FLHS are intricately linked to oxidative stress and chronic inflammation. The organic acids, peptides, and probiotic metabolites generated during the fermentation process can enhance antioxidant capacity, decrease levels of reactive oxygen species, and inhibit the activation of inflammatory signaling pathways, thereby mitigating liver cell damage and inflammatory responses [46]. Concurrently, fermentation products can ameliorate inflammatory damage mediated by the gut-liver axis by modulating the composition of the gut microbiota, enhancing gut barrier function, and reducing the translocation of endotoxins (such as lipopolysaccharides) into the portal vein circulation. Additionally, fermented FLL can diminish hepatic lipid accumulation by downregulating the expression of lipogenic genes, including *SREBP1* and *FAS*. In summary, the fermentation process not only enhances the bioavailability of FLL active components but also exerts synergistic effects through multiple mechanisms, including antioxidant and anti-inflammatory actions, modulation of the gut microbiota, and improvement of lipid metabolism, thereby offering significant therapeutic benefits for the treatment of fatty liver disease [47].

## 5. Conclusions

In summary, the inclusion of FLL fermentation products in the diet of layers affected by FLHS effectively inhibited hepatic fatty acid synthesis via the *NR1H3-ACC/FAS* pathways, thereby reducing fat accumulation and lipid metabolism disorders. Additionally, supplementation with FLL fermentation products enhanced the antioxidant capacity of the layers. These findings indicate that dietary FLL fermentation products can indirectly alleviate FLHS in laying hens by enhancing antioxidant capacity and decreasing lipid peroxidation in tissues. Furthermore, the supplementation significantly improved the laying rate and egg weight while reducing the eggshell breakage rate. This study provides a comprehensive analysis of the effects of dietary FLL fermentation products on hens with FLHS, with a focus on fatty acid metabolism, antioxidation, and related gene expression responses. Based on various indicators, the optimal dosage of dietary FLL fermentation products was determined to be 0.1% under the conditions of this experiment.

## Figures and Tables

**Table 1:** Diets composition and nutrient levels (air-dry basis).

Items	Basal Diet	High-Energy Diet
Ingredients (%)		
Corn	65.00	61.00
Soybean meal	22.00	22.00
Limestone	8.00	8.00
Soybean oil	−	4.00
Premix 1	5.00	5.00
Total	100	100
Nutrient levels 2		
Metabolizable energy (MJ/kg)	10.96	11.92
CP (%)	15.50	14.52
Ca (%)	3.52	3.52
AP (%)	0.43	0.43
Lys (%)	0.78	0.78
Met (%)	0.39	0.39
Met + Cys (%)	0.67	0.67

CP = Crude protein; AP = Available phosphorus. 1 The premix provided the following per kg of diet: VA 7500 IU, VD3 2500 IU, VE 35 mg, VK3 1 mg, VB1 1.5 mg, VB2 4 mg, VB6 2 mg, VB12 0.02 mg, nicotinic acid 30 mg, folic acid 0.55 mg, pantothenate 10 mg, biotin 0.16 mg, choline chloride 420 mg, The basal diet premix contains 2% protein, while the high-energy diet premix contains 8.4% protein. 2 The soybean meal used in the experimental diets contained 46% crude protein, crude protein was a measured value and others were calculated values.

**Table 2:** The accession number of target genes, forward and reverse of primer sequence, product size.

Gene	Accession NO.	Sequences (5′ to 3′)	Product Size (bp)
*LPK*	NM_204426.2	F: CAGCGTGGTATTGTGGGTCT R: TCACCACGATCACCCTTCAC	245
*MTTP*	NM_001109784.3	F: ATCCCATTAGCGTCGTG R: GCTGAACTCCATCCCTCC	137
*ACC*	NM_205505	F: AATGGCAGCTTTGGAGGTGT R: TCTGTTTGGGTGGGAGGTG	136
*FAS*	NM_001199487.2	F: ACAAAGCACTCGGTTTGGAG R: TGACTCGCAATGTTCACACC	113
*SCD*	NM_204890.2	F: CGGATGCAGACCCTCACAAT R: GGGCTTGTAGTATCTCCGCTG	164
*NR1H3*	NM_204542.3	F: CATGCGGGAGCAGTATGTTCT R: TGCTTCGCGGTTATTAGGGT	262
*SREBF1*	NM_204126.3	F: GCCCTCTGTGCCTTTGTCTTC R: ACTCAGCCATGATGCTTCTTC	130
*ChREBP*	NM_001398177.1	F: AGCCCAGCATTAAGGTCAGC R: CGGATGATGACGCCGAAGAT	283
*β-Actin*	NM_205518.2	F: GTATGTGCAAGGCCGGTTTC R: TCTGGGCTTCATCACCAACG	135

*LPK* = L-type pyruvate kinase; *MTTP* = Microsomal triglyceride transfer protein; *ACC* = Acetyl-CoA carboxylase; *FAS* = Fatty Acid Synthase; *SCD* = Stearoyl-CoA desaturase; *NR1H3* = Nuclear receptor subfamily 1 group H member 3; *SREBF1* = Sterol regulatory element binding protein 1; *ChREBP* = Carbohydrate response element binding protein.

**Table 3:** Effect of different dietary treatment groups on egg production performance of laying hens during late laying period.

	Dietary Groups 1					
Project	I	II	III	IV	SEM	*p*-Value
laying rate (%)	90.90 a	89.17 b	89.56 ab	90.64 a	0.24	<0.05
egg weight (g)	60.49 c	59.94 d	63.07 a	62.64 b	0.115	<0.001
egg breaking rate	0.66 b	3.21 a	0.26 b	0.62 b	0.183	<0.001

1 I: basal diet group, II: high-energy diet group, III: high-energy diet + 0.05% FLL fermentation product group, IV: high-energy + 0.1% FLL fermentation product group. a, b, c, d Values within a row with different superscripts differ significantly at *p* < 0.05.

**Table 4:** Effects of different dietary treatment groups on serum biochemical indices of laying hens.

	Dietary Groups 1					
Project	I	II	III	IV	SEM	*p*-Value
HDL-C (mmol/L)	0.90	0.76	0.84	0.81	0.031	0.457
LDL-C (mmol/L)	1.52 c	3.89 a	2.93 b	1.92 c	0.291	<0.001
TG (mmol/L)	12.02 b	18.91 a	12.95 b	10.54 b	1.057	<0.05
TCH (mmol/L)	2.45 b	4.04 a	2.71 b	2.67 b	0.238	<0.05
ALT (U/L)	2.88 c	6.30 a	4.91 b	3.70 c	0.416	<0.001
AST (U/L)	29.41	31.82	30.84	26.69	0.832	0.128

HDL-C = High-density lipoprotein cholesterol; LDL-C = Low-density lipoprotein cholesterol; TG = Triglyceride; TCH = Total cholesterol. 1 I: basal diet group, II: high-energy diet group, III: high-energy diet + 0.05% FLL fermentation product group, IV: high-energy diet + 0.1% FLL fermentation product group. a, b, c Values within a row with different superscripts differ significantly at *p* < 0.05.

**Table 5:** Effect of different dietary treatment groups on serum antioxidant indices of laying hens in late laying period.

	Dietary Groups 1					
Project	I	II	III	IV	SEM	*p*-Value
T-SOD (U/mL)	107.56 a	96.96 b	108.94 a	116.96 a	2.536	<0.05
GSH-Px (U/mL)	504.20	442.35	446.05	476.30	18.350	0.665
CAT (U/mL)	0.84	0.65	0.83	0.89	0.081	0.791
MDA (nmol/mL)	5.14 b	11.71 a	4.38 b	3.24 b	1.197	<0.05
T-AOC (U/mL)	12.99 a	7.03 b	12.29 a	12.54 a	0.817	<0.05

T-SOD = Total superoxide dismutase; GSH-Px = Glutathione peroxidase; CAT = Catalase; MDA = Malondialdehyde; T-AOC = Total antioxidant capacity. 1 I: basal diet group, II: high-energy diet group, III: high-energy diet + 0.05% FLL fermentation product group, IV: high-energy diet + 0.1% FLL fermentation product group. a, b Values within a row with different superscripts differ significantly at *p* < 0.05.

## Data Availability

The original contributions presented in this study are included in the article. Further inquiries can be directed to the corresponding author.
